# Single-shot X-ray absorption spectroscopy at X-ray free electron lasers

**DOI:** 10.1038/s41598-023-44196-2

**Published:** 2023-10-24

**Authors:** Marion Harmand, Marco Cammarata, Matthieu Chollet, Andrew G. Krygier, Henrik T. Lemke, Diling Zhu

**Affiliations:** 1 IMPMC, Sorbonne Université, UMR CNRS 7590, MNHN, 75005 Paris, France; 2 Institut de Physique de Rennes, UMR UR1-CNRS 6251, Université de Rennes 1, 35042 Rennes, France; 3 LCLS, SLAC National Accelerator Laboratory, Menlo Park, CA 94025 USA; 4 Lawrence Livermore National Laboratory, 7000 East Ave, Livermore, CA 94550 USA; 5 SwissFEL, Paul Scherrer Institut, Villigen, 5232 Switzerland; 6 Present Address: European Synchrotron Radiation Facility, Grenoble, France

**Keywords:** Characterization and analytical techniques, Free-electron lasers

## Abstract

X-ray Absorption Spectroscopy (XAS) is a widely used X-ray diagnostic method for studying electronic and structural properties of matter. At first glance, the relatively narrow bandwidth and the highly fluctuating spectral structure of X-ray Free Electron Lasers (XFEL) sources seem to require accumulation over many shots to achieve high data quality. To date the best approach to implementing XAS at XFEL facilities has been using monochromators to scan the photon energy across the desired spectral range. While this is possible for easily reproducible samples such as liquids, it is incompatible with many important systems. Here, we demonstrate collection of single-shot XAS spectra over 10s of eV using an XFEL source, with error bars of only a few percent. We additionally show how to extend this technique over wider spectral ranges towards Extended X-ray Absorption Fine Structure measurements, by concatenating a few tens of single-shot measurements. Our results pave the way for future XAS studies at XFELs, in particular those in the femtosecond regime. This advance is envisioned to be especially important for many transient processes that can only be initiated at lower repetition rates, for difficult to reproduce excitation conditions, or for rare samples, such as those encountered in high-energy density physics.

## Introduction

X-ray Absorption Spectroscopy (XAS) is an extremely powerful diagnostic for simultaneously probing the electronic structure and the local atomic arrangement. XAS is a widely used technique and its user community is well established among synchrotron users spanning a wide variety of research topics such as fundamental condensed matter physics, materials science, plasma physics, biology, geoscience, chemistry, industrial applications and cultural heritage^1^. More recently, the development of time-resolved XAS, often coupled to pump-probe techniques, has allowed tracking of the ultrafast interplay between electron and atomic motion, or opening new perspectives for understanding complex processes^2, 3^.

Several X-ray Free Electron Laser (XFEL) facilities operating around the world are capable of routinely producing beams with sub 10 femtosecond (fs) pulse width^4^ tunable photon energy and extremely high intensity (up to 10$^{20}$ W/cm$^{2}$). As a consequence, it is possible to apply traditional x-ray synchrotron techniques to a wider range of systems not previously accessible. For example, a concerted effort of many research groups has paved the way to the development of serial femtosecond crystallography based on fs x-ray diffraction snapshots of thousands of micron size crystals^5^. Ultrafast single-shot x-ray diffraction measurements of laser compressed samples are also now commonly performed at XFEL facilities allowing pump—probe measurements of transient phases and phase transition mechanisms^6, 7^. In addition, the high brightness of the XFEL pulse often enables (quasi-)single-shot measurements, ensuring better statistics, higher reliability of the data and allowing wide ranges of conditions to be scanned during an experiment.

As far as XAS is concerned, most implementations (at synchrotrons and XFELs) use an x-ray monochromator to sequentially scan the X-ray photon energy over the range of interest. This approach is very well suited for dilute samples or for samples that can be reliably excited at high repetition rates (>10 Hz). At XFELs, the Self-Amplified Spontaneous Emission (SASE) process, currently used in all hard x-ray facilities, results in high beam brightness but intrinsically highly fluctuating features. For instance, the pulse arrival time, the pulse intensity, the spectral energy and the spectral features themselves all vary strongly from shot-to-shot. Specific developments have optimized data collection and analysis of these shot-to-shot fluctuations, notably the intensity^8^ and the laser to x-ray arrival time jitter^9^. These developments have made it possible to average over thousands of repeatable shots per time-delay and study a variety of systems from short lived excited states in organometallic molecules^10, 11^ to proteins in solutions^12, 13^, as well as 3d metal foils^14, 15^.

It should be stressed that, for all works mentioned above, the time dependent XAS has been measured by averaging thousands of laser-pump/xray-probe pulse pairs per desired x-ray photon energy, which is not currently possible for experiments with samples available in small quantities, or when using “pump-pulses” with $\sim$0.1–0.001 Hz repetition rates (such as high-power lasers). It also imposes strong experimental restrictions, limits the variety of conditions that can be probed during a standard allocation of experimental time and requires post-experiment sorting and over-accumulation of data. If such an experiment could be performed in a single- or few-shot mode, it would be a much more efficient process that would allow a much larger phase space to be explored during limited periods of experimental access, as well as to enabling fs time-resolved Extended X-ray Fine Absorption Spectroscopy (EXAFS).

To overcome these limits, single-shot XAS capabilities have been developed at synchrotrons^16–19^, at XFELs^20–22^, and at facilities using optical laser-driven x-ray sources^23, 24^. These developments are all based on a dispersive method that allows an entire spectral range to be collected at once, using dispersive optics and position sensitive detectors. A second important aspect consists in enabling high-quality normalisation by measuring the so-called $I_0$ reference spectra. At synchrotron facilities, major effort was put into stabilizing the x-ray beam and optics to ensure negligible deviation between the transmitted spectra $I_T$ and the reference spectra $I_0$. In the case of XFELs^20–22^, the shot-to-shot fluctuations prohibit using a similar approach. Therefore, dispersive XAS setups and $I_0$ normalisation were performed by either using a spatially resolved spectrometer^20, 21^ that allows spatial discrimination between the transmission and the reference spectra, or by using two spectrometers^22^ to independently measure the transmitted and the $I_0$ spectra. The first method is fairly limited as it can only work with unfocused or partially focused geometry and assumes a homogeneous distribution of the XFEL spectra over its spatial dimension. Coupled with a specific data treatment, both methods allow accumulation over tens of shots to reach several percent signal to noise ratio. Unfortunately, high-quality single-shot XAS measurement, with under few percent signal-to-noise and <1eV spectral resolution could not be demonstrated due to the intrinsically sharp spectral features that vary from shot-to-shot. As a consequence, these XAS techniques require data accumulation and assuming sufficiently identical shot-to-shot experimental conditions for averaging, which in the best case is not ideal and can also place undesirable constraints on the experimental conditions and configurations^22^.

In this work, we investigate the ability to perform two regimes of XAS: X-ray Absorption Near Edge Spectroscopy (XANES) and up to $\sim$ 7Å$^{-1}$ EXAFS. We demonstrate a new and original approach to performing ultrafast dispersive XANES in a truly single-shot as well as multi-shot ($\sim$ 10) EXAFS measurements at XFEL facilities. We address the highly stochastic structure of the x-ray spectrum with multiple advances. First, we pursued our development of identical two spectrometers to monitor both the incident reference spectra and the transmitted spectra and hence allow a shot-to-shot normalisation of the spectra in a dispersive geometry. Second, we have developed an alignment procedure to address the intrinsic spatial chirp and optical transport effects of the XFEL pulse. Third, we have developed an iterative spectral registration optimization that allows us to accurately characterize the XAS signal with the intrinsically stochastic XFEL spectrum.

## Experimental approach

**Figure 1:**
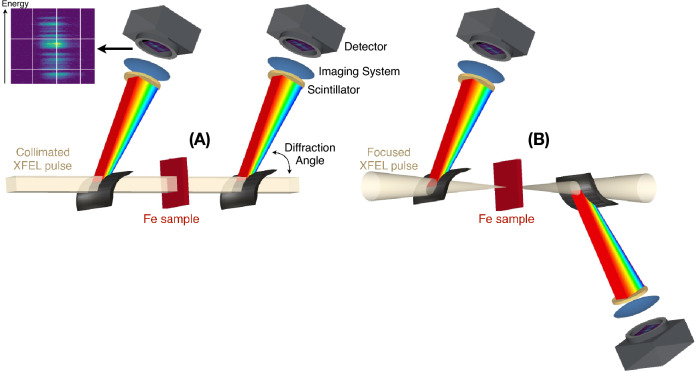
Energy dispersive experimental setup at LCLS with the unfocused collimated (**A**) and the focused (**B**) XFEL beam. Two identical spectrometers allow simultaneous measurement of the incident and transmitted spectra.

The presented experiment was performed at the XPP instrument of the LCLS^25, 26^. Dispersive XAS measurements using unstable x-ray sources require normalizing a measured transmitted spectra $I_{T}$(E) by the incident spectra $I_{0}$(E) for every single x-ray pulse. The energy dispersive experimental setup (see Fig. 1) consists of two identical spectrometers^22, 27^ in a reference—transmission geometry. The first spectrometer, placed upstream of the sample, collects the reference source spectrum while the second spectrometer, placed downstream of the sample, collects the transmitted spectrum. This experimental setup, combined with a dedicated and careful spectrometer alignment procedure, an advanced data analysis technique and tuning of the XFEL spectral properties allows us to overcome the limitations induced from XFEL typical shot-to-shot spectral and spatial variations.

### X-ray spectrometers co-alignment

Dispersive XAS was already attempted at XFELs using different spectrometer designs^20–22^, all aiming at measuring $I_{T}$(E) and $I_{0}$(E) simultaneously as well as correcting any perturbations that would affect the transmitted spectra beyond the sample effect itself. While successive corrections were acquired and applied in order to correct from beamline optical transport effects or experimental drifts, the shot-to-shot fluctuations between the incident and transmitted spectra remained problematic. In our configuration, the Bragg angle is set by the angle between the collimated XFEL beam and the local tangent of the bent crystal, which varies due to its curvature. This combination creates a correlation between photon energy and position in the XFEL beam (along the dispersion direction of the spectrometer, vertical in Fig. 1A). In other words, the dispersive mechanism of the single-shot spectrometer maps different spatial positions of the XFEL beam profile and the quality of the absorption spectra therefore relies critically on the relative alignment and calibration of the spectrometer pairs along the XFEL beam position. An implicit assumption of previous studies is that the spectral content of the incident beam is spatially uniform. This however, is not always the case for the SASE FEL pulses. Past observation have shown that spectra sampled from different part of the beam can be significantly different. This correlation between the spectral content and the spatial coordinate of the XFEL pulse profile is called ”spatial chirp” and might be overlooked at first but will ultimately limit the achievable signal-to-noise if not considered.

We minimize the impact of spatial chirp on our measurements in two ways. First, we chose the vertical scattering geometry in which the magnitude of the spatial chirp is expected to be the lowest due to the horizontal dispersion elements in the accelerator^28^. Second, we ensure that each photon energy is measured from a closely neighboring spatial part of the beam profile on both spectrometers by aligning them to the same vertical location in the XFEL beam. This is accomplished by rotating the crystal angle of the downstream spectrometer relative to the XFEL beam (see the Supplementary Information SI Fig. 1), and thereby scanning different mappings of Bragg angles to beam positions. When the two spectrometers are perfectly aligned and diffracting photons from the same part of the XFEL beam, an intensity drop is observed in the second spectra and caused by extinction from the crystal of the incident upstream spectrometer. Once the extinction position is recorded, the second spectrometer is slightly misaligned (see supplementary material) to allow recording the transmitted spectra. This alignment procedure significantly improves the quality of the measurement as shown in figures SI Figs. 2 and 3 of the supplementary material. Therefore it should be performed for each beamline configuration and FEL operation mode that might affect the XFEL chirp distribution over its beam profile.

### Data analysis

The spectrometer alignment described above is a necessary, but insufficient step for our purposes. Remaining small variations between the spectrometers such as crystal curvature, crystal mounting to substrate, etc. cause variations in the spectral and spatial mapping onto the detector, preventing perfectly identical spectral measurements. However, a unique and systematic spatial-spectral dispersion for each spectrometer can be corrected for. For that purpose it is possible to define a 2D image transformation function $\mathcal {T}_{2D}$ to match the downstream and upstream spectrographs to each other. This function $\mathcal {T}_{2D}$ is derived from the average of a few tens of pulses acquired with no sample and is then applied to the absorption spectra with the sample inserted. $\mathcal {T}_{2D}$ consists of 2D affine transformations such as rotation, translation and shear. In an iterative process, the difference between the two spectrometer images is minimized by adjusting the transformation parameters of $\mathcal {T}_{2D}$ (see Method and Supplementary material). The quality of this iterative procedure is estimated by calculating the absorbance *A*, that should equal 0 at all energies in absence of a sample. $A = -log(T) = -log \left( \frac{I_{T}(E)}{I_{0}(E)} \right)$, with $I_{T}(E)$ and $I_{0}(E)$ being the integration of the 2D images, perpendicular to the dispersive direction. The minimized standard deviation $\sigma$ of the no-sample absorbance is used to quantify the noise associated with our measurements. We also found that it is necessary to record the no sample reference spectrographs shortly (ideally tens of minutes) before inserting the sample for actual data collection.

### XFEL spectral optimization for XAS

We have investigated various XFEL operation modes to directly modify the XFEL spectral characteristics and optimize the single-shot absorption spectra quality. For single-shot XAS, the two primary goals are to minimize the effect of the valleys in the spiky x-ray spectrum, which turns inherently into noisy part of the absorption spectra and to increase the XFEL bandwidth to the $\sim$ 50-100 eV required for XANES. We therefore investigate several non-standard modes that could potentially broaden the spectrum and its spiky features. In the normal operation mode, the SASE bandwidth is determined largely by the undulator gain length, and the resulting spectral full widths are typically 0.2–0.3$\%$, or 15–20 eV at 7 keV^29^. One way to increase the overall bandwidth of the x-ray pulses is to increase the energy-chirp of the electron bunches^30, 31^. In practice, the over-compression mode, with electron bunch head-tail switches in the compressor chicane of the accelerator^29, 32^, produces a reversed energy correlation along the length of the electron bunch (lower energy at the tail side) known as chirp. Linac structure wakefields downstream of the compressor chicane introduce additional energy loss in the tail part of the electron bunch, which further enhances the amplitude of the existing energy chirp. By spreading the electron energy, different slices of the bunch in the undulator can therefore lase independently at different photon energies leading to increased XFEL bandwidth^33^ but also ensure a minimal number of x-ray photons over the whole bandwidth. We understand the over-compressed mode to minimize the effect of the deep valleys in between the peaks in the spectrum. These valleys with almost zero photon numbers strongly degrade the signal-to-noise ratio at these energies in the spectrum. The choice of the over-compressed mode doesn’t maximize the intensity per pulse but rather increases the low-photon number parts of the spectrum, preventing overly deep valleys. It ensures the full bandwidth has sufficient photon flux for low-noise, single-shot measurements.

### X-ray absorption spectroscopy from a focused XFEL

**Figure 2:**
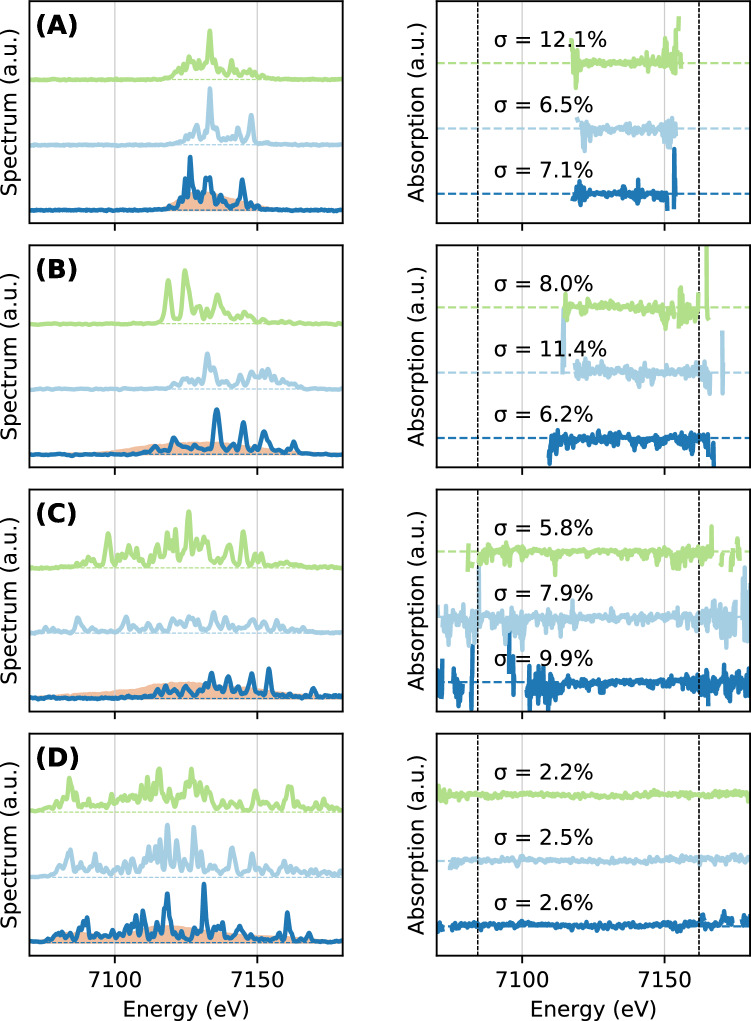
Left—Three consecutive single-shot XFEL spectra are displayed in dark blue, pale blue and green. They have been obtained for various tuning of the LCLS machine^33^ : low charge mode^54^ (**A**), low charge mode with large bandwidth (**B**) nominal mode full charge (**C**), overcompressed mode^29^ (**D**). The 1000-shot average spectra is shown in orange on the left panel. Right—Corresponding single-shot no-sample absorption spectra with their calculated standard deviation $\sigma$, showing the fluctuations around the average absorption value. This value is calculated for the spectral range shown by vertical dashed lines. The extraction of the absorption spectra is described in the method section.

Finally, we also evaluated the capability of measuring a single-shot XANES spectra in a focused beam geometry that is usually needed to reach x-ray focal spots smaller than $\sim$ 100 $\upmu$m. In this case, the two spectrometers are symmetrically positioned up and down stream of the beam focus. In order to preserve the spatial-energy mapping relation in the spectrogram, we needed to flip the scattering direction of the second spectrometer (see Fig. 1B). We used compound refractive lenses with 4 meters focal length and with the best focus set close to the sample position. The obtained XANES spectra are presented in Fig. 2 without and with sample (left and right respectively). This demonstration is particularly important as we expect this to be a more common experimental configuration. Indeed, most of the experiments only probe a small fraction of the excited sample as the probed region is usually required to be smaller than the pumped one and vary from few $\upmu$m to few hundreds of $\upmu$m. For example, the sample can be in the form of a 10 $\upmu$m scale diameter liquid jet^34^, or the uniformly excited region of the sample may be limited by the pump laser power and spot size to well below 100 $\upmu$m^35^. This is also particularly relevant for extreme high pressure and laser heated Diamond Anvil Cell studies that are usually performed with samples measuring a few tens of microns^16, 36^.

## Results

### No-sample x-ray absorption spectroscopy and quality assessment

Figure 2 shows spectra for four different machine operation modes : the low-charge mode (A), the enlarged bandwidth low-charge mode (B), the normal operation mode (C), and the over-compressed mode (D). Three random single-shot spectra are displayed for each of the four XFEL configurations. The acquisition is performed without a sample to assess the noise associated with our absorption measurement. A perfect spectrometer alignment and registration would yield zero absorption in the no-sample case and we use the standard deviation $\sigma$ of the measured absorption spectra as a metric for evaluating the quality of each XFEL mode. In the Fig. 2-Right, x-ray absorption spectra corresponding to each (A)–(D) XFEL spectra are calculated after applying the transformation function $\mathcal {T}_{2D}$ (see Suppl. Mat.). The function is optimized on previous shots as described above. In each plot, we write the $\sigma$ above the absorption curve of each shown spectra. The $\sigma$ is calculated in the range bound by the dashed vertical lines, which corresponds to the regions of interest for XANES measurements at the iron K-edge. As we can observe, the widely varying $\sigma$ shows the XFEL tuning strongly impacts the signal to noise ratio of the XANES measurement and therefore the quality of the single-shot data.

First, the fluctuating XFEL spectral spikes are clearly visible in the measured spectra (left panels of Fig. 2). The highly non-uniform distribution of the intensity is a challenge for normalization as for each shot there will be parts of the spectral range with very low intensity. Our previous work performing dispersive XANES measurements using XFEL radiation addresses the problem by averaging multiple shots in order to obtain a more uniform distribution of the reference spectra^20, 22^. In this experiment however, we overcome this challenge by first identifying and optimizing the XFEL operation mode to increase the spectral bandwidth and more importantly minimize the spectral regions with few photons, i.e. ensuring a minimum level of signal at each energy of the spectrum. This XFEL machine optimization has been performed by the accelerator experts of LCLS and consists optimizing different modules of the Linac that directly affect the properties of the electron bunch spectra and therefore the single-shot x-ray spectra.

In particular the ‘over-compressed’ mode allows us to extend the spectral range to almost 100 eV, more than 5 times of the typical SASE bandwidth, as shown in Fig. 2D. Here, the overcompressed mode systematically shows a standard deviation $\sigma$ below 0.05 and down to 0.02–0.03 in most of the cases. This implies ~2–3% noise associated with our single-shot absorption measurements. This mode has been identified as the most suitable for single-shot XANES measurements at LCLS. Other modes such as the low charge mode, with and without enlarging of the bandwidth or the nominal mode full charge (Fig. 2A,B,C respectively) produce between $\sim$ 4 and 10$\%$ noise in average. All data presented below have been obtained with the over-compressed mode.

### Fe K-edge x-ray absorption measurements

**Figure 3:**
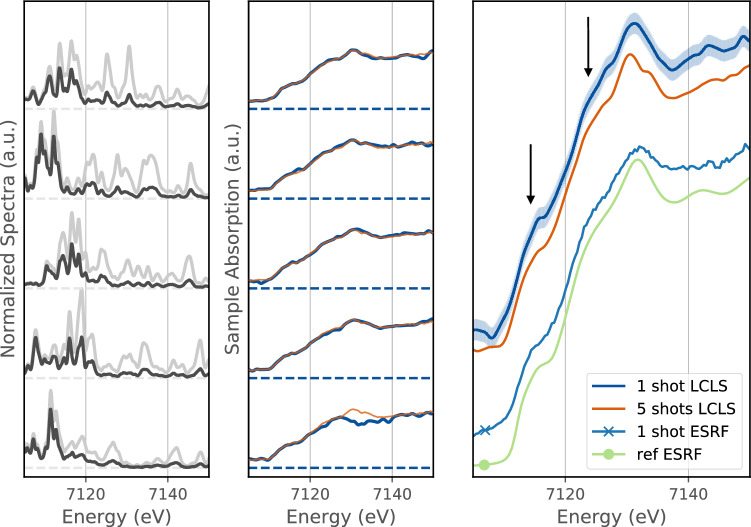
Left panels—XFEL spectra measured before (light gray) and after (black) the sample with the optimized FEL mode for 5 consecutive shots; Middle panels—Corresponding single-shot XANES spectra (blue line) and 5 shots averaged spectra (orange line); Right panel—comparison of different XANES spectra: a single-shot LCLS spectra (blue), 5 shots LCLS averaged (orange), a single bunch ESRF-ID24 spectra (blue with a cross) and a long time accumulation reference spectra from ESRF (light green with a full dot). The spectra are vertically shifted to improve the clarity of the figure. The two arrows are showing the pre-edge and shoulder distinctives features of the iron K-edge XANES.

To further assess the quality of the absorption spectra measured with the dispersive setup, we have measured absorption spectra of a 4 $\upmu$m thick iron foil (Goodfellow) and compared with dispersive data from ESRF^16, 17^. For comparison, the spectrum of a typical undulator source at a third generation synchrotron has a continuous spectrum with a minimum bandwidth of several hundred eV^16^. In Fig. 3, we show five consecutive spectra and their associated sample absorption, convolved with 0.5 eV smoothing resolution function. Here, the Fe K-edge is clearly observable in a single shot as well as the well identified pre-edge and shoulder features at 7.115 keV and 7.125 keV respectively^37^. Averaging over the five shots in Fig. 3 clearly shows an improvement of the signal to noise and highlights the stability of the single shot data. A single-shot and a long time accumulation XANES spectra taken at ESRF—ID24^17^ are also shown for comparison. The good agreement between ESRF and LCLS data confirms that high quality data can be obtained using an XFEL with one or a few shots despite the unstable, jagged structure of the x-ray spectrum produced by the SASE process.

**Figure 4:**
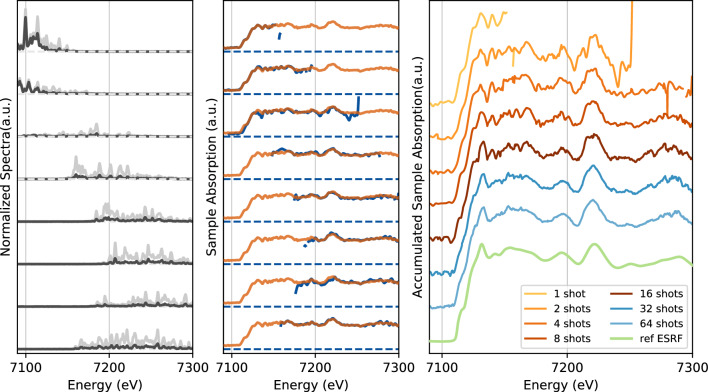
Left panels—XFEL spectra of the ‘best mode’ coupled with a controlled photon energy drift to enlarge the average spectral range. The two spectra from the incident and transmitted spectrometers are represented in gray and black respectively. Middle panels—Single-shots (blue line) and 10 shots averaged absorption spectra (orange line). Right panel—Large spectral range absorption spectra obtained for 1 to 64 accumulated shots compared with a long time accumulation spectra from ESRF (light green).

Despite the improved single shot spectral window ($\sim$100 eV) obtained with the optimized XFEL settings, an extended spectral range is necessary for certain applications^24, 38, 39^. This can be done by changing the central wavelength of the emitted XFEL spectrum (see Fig. 4Left). Note also that at LCLS at least, it can be done by the user in a fraction of a second, within 100-200 eV spectral range (larger changes require machine expert tuning). In addition, the spectral range of the Si membrane spectrometers intrinsically covers several hundreds of eV in this configuration, allowing up to 7Å$^{-1}$ wavenumber EXAFS spectrum to be recorded with minimal efforts and almost immediately. Such spectrometers can easily be modified to cover a larger spectral range with lower spectral resolution^27^. To test that our experimental approach was still valid in this case, we have changed the central energy by  100 eV over the data collection of several shots. In Fig. 4, accumulated spectra over a large spectral range are shown for 1, 2, 4, 8, 16, 32 and 64 shots and are again compared with a typical long-time-accumulation spectra from the ESRF synchrotron. Our results demonstrate that it is easily feasible to extend our XAS measurements towards EXAFS features that can be reconstructed with only a few shots, and that averaging over 10-20 shots allows access to detailed spectral features over an extended energy range.

### Fe K-edge x-ray absorption with focusing XFEL

**Figure 5:**
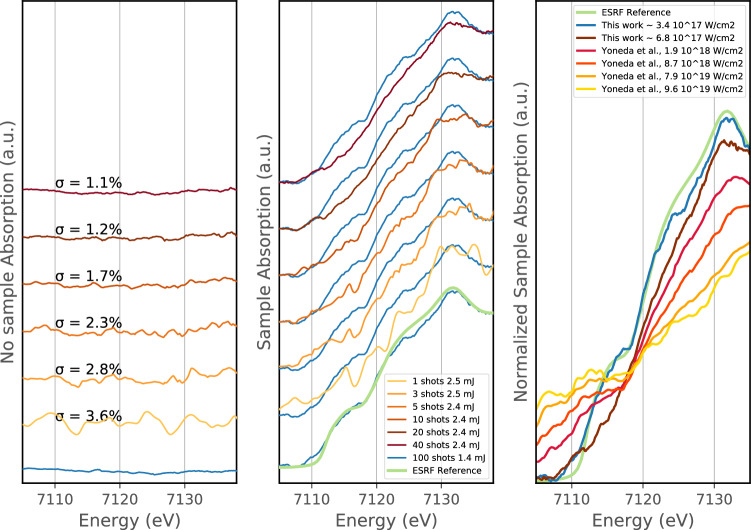
Left and Middle—Absorption obtained without a sample (Left) and with a 4 $\upmu$m Fe sample (Middle) for a focused XFEL. The XFEL spot size is estimated around 3 $\upmu$m. The number of accumulated shots is varying from 1 to 40 and the respective standard deviations of the absorption without sample $\sigma$ are varying from 3.6$\%$ to 1.1$\%$. On the Middle panel, the accumulated reference spectra from ESRF is also shown in light green and compared with an accumulation of 100 shots at low XFEL flux from the present experiment (in blue). Right panel—We overlay Yoneda’s results^15^ with our absorption spectra at high and low XFEL intensities (40 shots accumulated over fresh iron samples at 2.4 mJ and 100 shots at 1.4 mJ respectively). All results have been equally normalized by determining their normalization factors from their respective ambient condition iron XANES measurement, which were then applied to the high-intensity XFEL XANES spectra.

In Fig. 5Left and Middle, we show absorption spectra obtained for 1, 3, 5, 10, 20, 40 and 100 accumulated shots with a few micron focused XFEL beam. The single-shot noise varies between 3 and 5 $\%$ and still achieves around 1$\%$ noise after accumulation. We note that the fine spectrometer alignment procedure was not performed for the focused beam configuration, which induces a higher standard deviation for the single-shot measurement (between 3 to 5$\%$ noise to be compared to 2–3$\%$ previously—see Fig. 2). The quality of our XANES measurement in the focusing geometry could be easily improved by following the alignment procedure described above for the unfocused beam.

Figure 5Middle, shows XANES spectra sorted from bottom-to-top by increasing number of accumulated single-shots corresponding to the Fig. 5Left. In addition, the data are sorted as a function of the XFEL pulse energy to observe its effect on the XANES spectra. A XANES spectra obtained by accumulating up to 40 single-shots of 2.4 mJ pulse energy (in brown) is compared with a XANES spectra from an average of 100 shots at 1.4 mJ pulse energy (in blue). Compared to the reference spectra from ESRF (green curve), we observe that the XANES features are already modified at low beam energy (around 1.4 mJ, repeated blue curve) and the modification increases with increasing XFEL pulse energy. These modifications are due to irradiation by the probe pulse and demonstrate the need to attenuate the XFEL beam in this scenario. This can affect the photon statistics for high signal-to-noise ratios and could imply that increasing the number of accumulated shots is required. The XFEL intensity at which modifications to the XANES occur through self-irradiation is dependent on the XFEL parameters (photon energy, pulse energy, pulse duration, focal spot size) and the material studied and should be checked as part of any experiment. It cannot be safely assumed that the XFEL probe occurs before the material electronic density of states has been influenced^40^.

## Discussion

### XFEL non linear effects

The increased irradiance in the focused XFEL configuration causes changes to the Fe absorption spectra including loss of edge features at 7117 eV, 7125 eV, and 7132 eV and edge broadening (see Fig. 5). Similar changes can be associated with elevated temperature^22^, however this cannot explain our observations due to the timescale of our experiment. In our case, the XFEL pulse is acting both as a pump and a probe during its $\sim$50 fs pulse duration. This timescale is comparable to the electron thermalization timescale (up to several tens of fs^41^) and much faster than electron—ion thermalization can occur^42^. Our sample is therefore out-of-thermal equilibrium and our measurement in the focused geometry reflects the rapidly evolving electron distribution and density of states. Figure 5-Right shows our experimental data overlaid with the recent XANES measurements by Yoneda et al^15^. Their results show a significant decrease of the absorption above the edge as well as K-edge broadening and shifting due to the high-density excitation of the 1s electrons. Yoneda et al. interpret the observed XANES changes as saturable absorption of Fe but at higher irradiance ($10^{18-20}$ W/cm$^{2}$ vs. 4–7 $10^{17}$ W/cm$^{2}$ here), achieved by using a 7 fs XFEL pulse duration. In our data, the increase of the transmission above the Fe K-edge is consistent with Yoneda’s data and would correspond to the onset of saturable absorption near the irradiance threshold between $10^{17}$ and $10^{18}$ W/cm$^{2}$. However, our results do not show similar behavior below the edge, where the absorption increases in Yoneda’s et al. paper. As the XANES features below the edge are strongly related to the local electronic density of states, we expect that these effects are more difficult to observe in our experimental conditions, where we used a $\sim$50 fs pulse duration. Indeed, XANES may be even more sensitive to saturable absorption when the XFEL pulse duration is closer to the core-hole recombination timescale (estimated between $\sim$500 as and few fs for a K-shell core hole in iron) as it is the case in^15^. At the fs timescale, XANES is directly related to the ultrafast changes of the density of states and less affected by the electron thermalization at longer time scales. Proper evaluation of the XANES signal would require detailed knowledge of the XFEL temporal pulse shape and more advanced analysis tools like Density Functional Theory in the non-linear photoionization regime, which exceeds the scope of this work. Additionally, we note that the XANES spectra obtained by Yoneda et al. were accumulated over $\sim$ 100 shots selected by post hoc XFEL pulse intensity sorting. Our focused geometry requires accumulation over only a few shots to observe an ultrafast change of the XANES spectra upon XFEL irradiation.

### Perspectives on single-shot x-ray absorption spectroscopy at XFEL

The presented results show that with the present spectrometer design, alignment procedure, and optimized machine parameters, x-ray absorption spectra can be measured with a single XFEL pulse. The combination of over compression of the electron bunches with additional tuning of the ’spatial chirp’ of the spectra provided single-shot XANES spectra with a few percent noise. In this operation mode, the XFEL bandwidth corresponds to almost 2$\%$. It is important to note that this operation mode also implies a longer pulse duration of the XFEL, on the order of around 50 fs. The spectral resolution is given by the thin-bent crystal spectrometers, with $\sim$ 0.5 eV resolution in the chosen Si(220) configuration. Higher resolution can be achieved by adopting a different crystal reflection^27^. The tuning of the XFEL mode has been performed directly on the x-ray absorption spectra in order to minimize the fluctuations of the absorption, allowing a few percent noise in a single-shot across the primary energy range. Our experimental design sets the path for EXAFS and future finer XANES measurements such as pre-edge modifications of the Fe K-edge following spin transitions^43^, valence state quantification^44^ and reduction of iron in exotic high pressures phases^45^, usually requiring signal variations between few $\%$ and down to less than 0.5$\%$. Such typical features of the Fe K-edge should be observable in a single or few shots, enabling further studies under irreversible conditions at XFELs. We should mention this method is only possible when using a transmission geometry and is not suited for studies of diluted samples or extremely thin samples. These are usually studied using total fluorescence yield measurements. However these techniques require significant data accumulation. Here we demonstrate the possibility to provide single-shot XANES and to fully benefit from such exceptional diagnostic that can provide both the electronic and atomic structures with sub-eV spectral resolution.

Because electronic structure transformation is active on the fs timescale, ultrafast time-resolved XAS holds great potential for further understanding material properties at extreme conditions^3, 46^. This is especially true when studying, for example, spin transitions and oxidation in Fe minerals^44^, melting curves under high-pressure^22, 36, 47, 48^, anomalous heating process such as bond hardening or bond weakening^20, 49^, electron - ion relaxation^50, 51^ or ionisation potential depression in warm dense matter^52^. Shock-compressed matter or ultrafast laser irradiation are systematically destructive experiments and their reproducibility is extremely sensitive to laser parameters and stability, sample qualities, or even alignment procedures. To date, the approach to managing this issue has been to average XAS spectra taken at similar conditions by ensuring parallel in-situ measurements for post facto sorting. This was performed at the cost of large error bars and wasted data. The approach presented here puts an end to this critical experimental limitation^22^. Furthemor, studies employing strong magnetic field can also benefit from high-quality single-shot XANES and x-ray Magnetic Circular Dichroism (XMCD) measurements. Such an approach at XFELs could be further enhanced by the new possibilities with the MHz bunch trains at the European XFEL to probe ns dynamics with single-shot XANES after a single laser pump excitation as well as variable gap undulator and two-color XFELs to quickly reconstruct EXAFS spectra. The fs pulse duration of the XFEL also makes it possible to track unprecedented transient dynamics. Our results open promising perspectives for fs time-resolved fine XAS measurements on highly out of equilibrium systems.

## Methods

### Details on the experimental setup

Each spectrometer is composed of a 5 mm $\times$ 15 mm $\times$ 10 $\upmu$m thick (220) Si membrane crystal as an analyser and a YAG screen coupled with an optical microscope and a CCD camera to record the spectra on a shot-to-shot basis. Such spectrometer designs are regularly used at LCLS and have been developed by Zhu et al.. They are fully described in reference^27^. At 7.1 keV and for a 50 mm radius of curvature, the spectral resolution is $\sim$0.5 eV, well below the core hole lifetime broadening of 1.25 eV for the iron K-edge^53^. The spectral range covered by the Si (220) spectrometer is $\sim$ 200eV, allowing the XFEL energy to be changed in order to perform spectral-range-limited EXAFS without changing the angle, nor realigning the spectrometers. The transmission of each thin Si crystal membrane is around 62$\%$ at 7.1 keV. The spectrometers were placed $\sim$ 200m from the source. At the sample position, the XFEL beam size varied from $150 \times 800$ $\upmu$m in the unfocused case (defined by slits) down to few $\upmu$m size when focusing. The focusing geometry using CRL lenses to focus the x-ray beam. In this case the second spectrometer is flipped vertically in order to keep the 2 systems identical, i.e. the second spectrometer must select the same energy from the same part of the beam as the first one which is reversed due to converging and diverging beam.

### Alignment of spectrometers

Both Si crystals membranes were first aligned by scanning their vertical and horizontal positions and centered on the beam. A fine relative alignment is performed by online minimisation of the no-sample absorption spectra fluctuations when scanning the Bragg angle of one of the spectrometer to match the other spectrometer alignment. This procedure aims at selecting the same spectral component of the XFEL from the same spatial position in the beam. This is a relative alignment and one could equally scan the downstream or upstream spectrometer. SI Fig. 1 in the supplementary material shows the integrated intensity of the downstream spectrometer while scanning the Bragg angle of the upstream spectrometer. We notice a sharp extinction of the signal at $-26.96$ degrees, that corresponds to the expected wavelength extinction at best alignment. To avoid complete extinction of the signal and poor quality measurements, we choose an optimized Bragg angle, slightly away from this extinction and at a maximum detected intensity. This position corresponds to the best standard deviation and is indicated by an arrow in the supplementary material.

### Analysis

The Python analysis code is freely available at https://github.com/marcocamma/dispersiveXanes. It includes tools to read and analyze the data as well as the scripts used to produce the figure of this manuscripts. SI Fig. 2 and SI Figure 3 in the supplementary materials shows 2D images of the two spectrometers and their corresponding integrated spectra. We also show the difference of the 2D images and the ratio of the spectra with their calculated standard deviation in the considered spectral range. SI Fig. 2 shows the results for a single-shot before image transformation while SI Fig. 3 shows the same single shot data but after data treatment. In the figures, we also overlay both spectra (red and blue respectively) to underline their differences before and after image transformation. Once the image transformation is applied to the data, we can observe that the 2 spectra are very well matched. This analysis results in a significant improvement of the absorption fluctuations. For this specific shot, the standard variation $\sigma$, with no sample inserted in the beam, varies from 0.71 to 0.04 after data treatment. We also underline that the data treatment is performed on a finer pixel grid than the actual spectrometer resolution. For that reason, all presented data are smoothed with a 0.5 eV resolution.

### Supplementary Information

Supplementary Figures.

## Data Availability

The datasets generated and/or analysed during the current study are available in the dispersiveXanes/figures/figures_v2/data/ repository, https://github.com/marcocamma/dispersiveXanes.
