# Supplementary material for: Single-shot X-ray absorption spectroscopy at X-ray free electron lasers
Source: Sci Rep. 2023 Oct 24;13:18203. doi: 10.1038/s41598-023-44196-2 (PMC10598033; doi:10.1038/s41598-023-44196-2)
Supplement: Supplementary file 1 — Supplementary Figures. [file 41598_2023_44196_MOESM1_ESM.pdf]

Supplementary Material for Single-shot X-ray Absorption Spectroscopy at X-ray Free Electron Lasers

M. Harmand, M. Cammarata, M. Chollet, A. Krygier, H.T. Lemke, D.Zhu

August 28, 2023

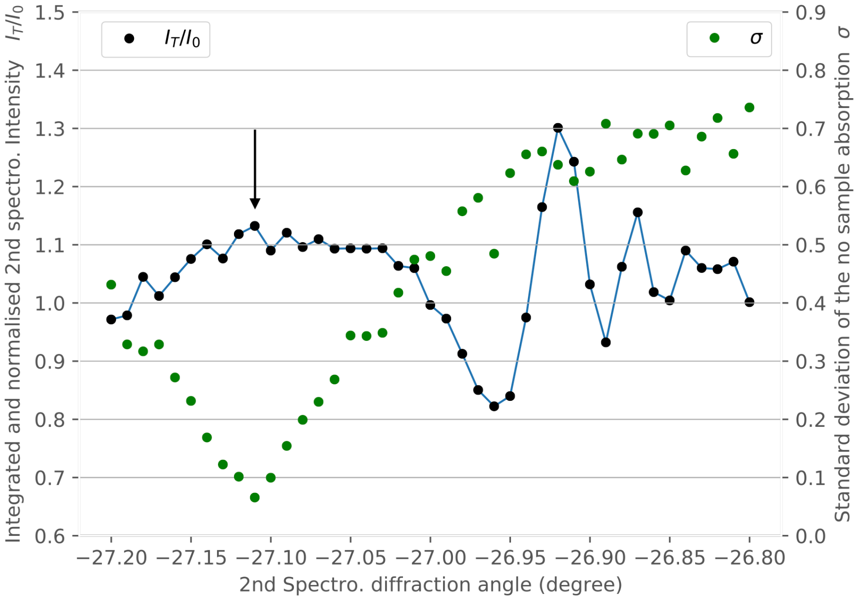

Figure 1: Normalized intensity of the transmitted spectra in function of the diffraction angle on the Si membrane to optimize the respective alignment of the two spectrometers. The final crystal angle for the spectral measurement was chosen to be just next to the angle where we have maximal intensity loss and where the standard deviation is minimal. This position is shown by an arrow in the figure

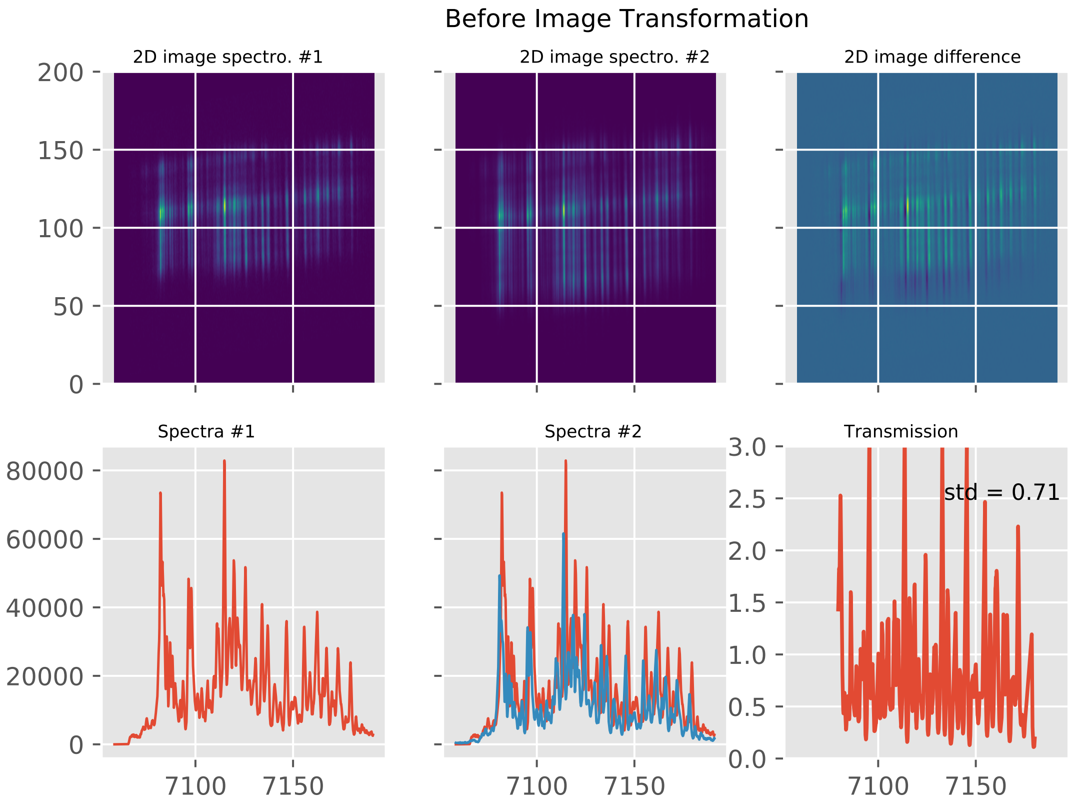

Figure 2: Data set of a single shot before the image transformation data treatment. Left column - 2D images and integrated spectra (in red) of the upstream spectrometer. Middle column - 2D images and integrated spectra (in bleu) of the dowstream spectrometer . In addition, the upstream spectra is overlaid in red. Right column, difference of the 2D images (top) and transmission calculated from the ratio of the integrated spectra (bottom). The calculated standard deviation σ of the transmission is 0.71. The color code associated with the 2D images indicates a photon intensity in arbitrary unit.

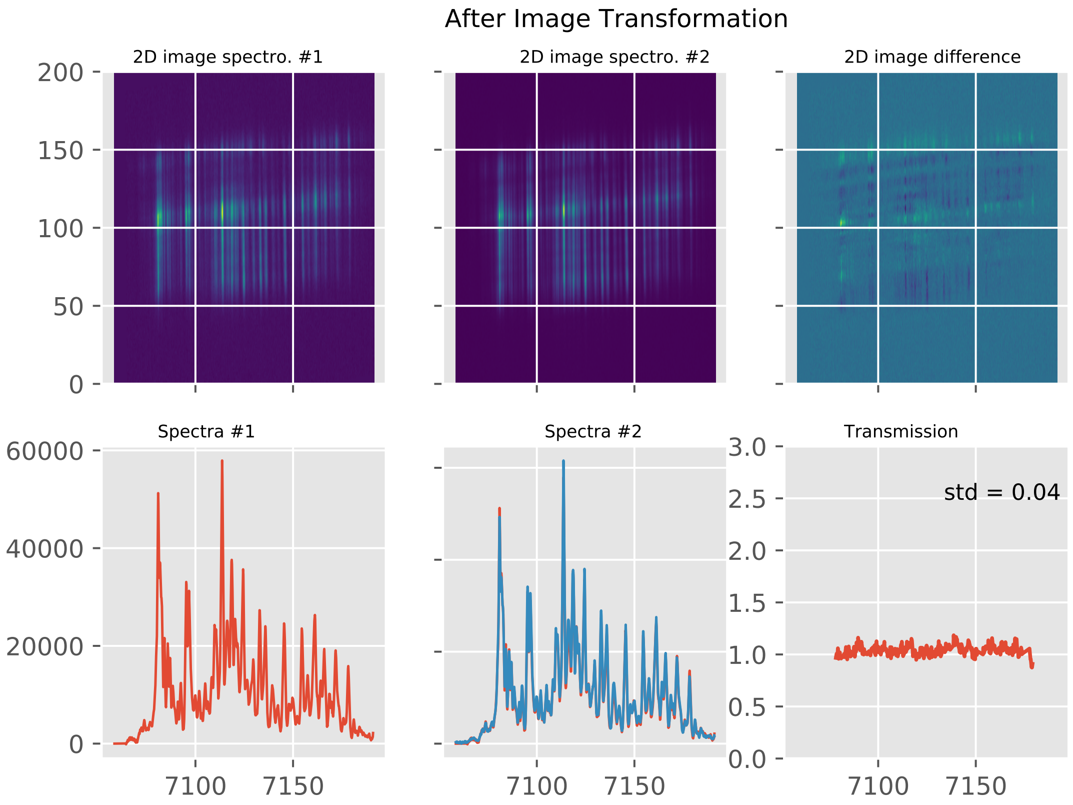

Figure 3: Data set of a single shot after the image transformation data treatment. Left column - 2D images and integrated spectra (in red) of the upstream spectrometer. Middle column - 2D images and integrated spectra (in bleu) of the dowstream spectrometer . In addition, the upstream spectra is overlaid in red. Right column, difference of the 2D images (top) and transmission calculated from the ratio of the integrated spectra (bottom). The calculated standard deviation σ is 0.04. The color code associated with the 2D images indicates a photon intensity in arbitrary unit.
